# Prospective Study of Glycated Hemoglobin and Trajectories of Depressive Symptoms: The China Health and Retirement Longitudinal Study

**DOI:** 10.14336/AD.2018.0410

**Published:** 2019-04-01

**Authors:** Haibin Li, Anxin Wang, Wei Feng, Deqiang Zheng, Qi Gao, Lixin Tao, Jin Guo, Xiaonan Wang, Xia Li, Wei Wang, Xiuhua Guo

**Affiliations:** ^1^Department of Epidemiology and Health Statistics, School of Public Health, Capital Medical University, Beijing, China.; ^2^Beijing Municipal Key Laboratory of Clinical Epidemiology, Capital Medical University, Beijing, China.; ^3^Greenwood Medical Company, 300 Highway Burwood, Melbourne, Melbourne, Victoria, Australia.; ^4^Department of Mathematics and Statistics, La Trobe University, Victoria, Australia.; ^5^Global Health and Genomics, School of Medical Sciences and Health, Edith Cowan University, Perth, Western Australia, Australia.

**Keywords:** Trajectory, depressive symptoms, glycated hemoglobin

## Abstract

The longitudinal association between glycated hemoglobin (HbA_1c_) and different courses of depressive symptoms is understudied. This study aimed to identify different trajectories of depressive symptoms and investigate the relation of HbA_1c_ with the risk of increasing and high-stable depressive symptoms. In the China Health and Retirement Longitudinal Study, depressive symptoms were measured using the 10-item Center for Epidemiological Studies-Depression scale in three visits (years: 2011, 2013 and 2015) among 9804 participants (mean age 60.0 ± 9.0 years). Group-based trajectory modeling was used to identify trajectories of depressive symptoms. HbA_1c_ was measured at baseline and categorized five groups according to the respective quintile. Multinomial logistic regression was fitted to examine this relationship. Four distinct trajectories of depressive symptoms were identified: low symptoms (n=6401, 65.29%); decreasing symptoms (n=1362, 13.89%); increasing symptoms (n=1452, 14.81%); and high symptoms (n=1452, 14.81%). Adjusting for demographic, health-related, and cognitive factors, the risk ratio (95% confidence interval) pertaining to the highest HbA_1c_ (Quintile 5) for decreasing, increasing, and high symptoms of depression versus low symptoms was 1.01 (0.82-1.25), 1.12 (0.92-1.36), and 1.39 (1.04-1.86) compared with the lowest HbA_1c_ (Quintile 1), respectively. We observed a J-shaped relationship between HbA_1c_ and high depressive symptoms, with the lowest risk at a HbA_1c_ concentration of 5.0%. In summary, in this large population-based cohort, high levels of glycated hemoglobin concentrations were associated with a higher risk of increasing and high-stable symptoms of depression.

Depression symptoms are highly prevalent and impact the quality of life of those struggling from them [1]. Especially, in China, the prevalence of depressive symptoms among the elderly has been reported to be up to 22.7% [2]. Moreover, depressive symptoms that increase over time are also associated with a range of adverse outcomes, including cardiovascular diseases [3, 4], dementia [5] and all-cause mortality [6]. Understanding risk factors attributed to depressive symptoms is a major public health priority; however, potential risk factors for depression symptoms remain unclear.

Several cohort studies [7-9] and meta-analysis [10-12] have revealed that depressive symptoms were associated with a high risk of diabetes mellitus. Additionally, a recent meta-analysis also shows that diabetes mellitus increased the risk of depression by 25% [13]. This suggests that the association between depressive symptoms and diabetes mellitus is more complex and may be bidirectional[8]. However, in most studies, depressive symptoms are assessed at a single point time (at baseline), and hence these studies may not have fully captured the longitudinal pattern of depressive symptoms.

Glycated hemoglobin (HbA_1c_) is considered as the gold standard measurement of glycemic control for diabetes mellitus patients, reflecting glucose exposure over the previous two to three months[14]. Previous studies found HbA_1c_ is associated with depressive symptoms [15, 16]. Furthermore, these studies neglect the relapsing and remitting nature of depressive symptoms. However, very few studies have provided information that describes the relationship between HbA_1c_ and the longitudinal pattern of depressive symptoms in the Chinese population.

Therefore, the aims of this study were to use repeated measures of depressive symptoms in three visits from China Health and Retirement Longitudinal Study (CHARLS), a nationwide representative cohort study, to identify the different trajectories of depressive symptoms, and investigate a possible link between HbA_1c_ and the trajectory of depressive symptoms. We hypothesize that high levels of HbA_1c_ is associated with increasing and high-stable depressive symptoms.

## MATERIALS AND METHODS

### Study design and population

CHARLS is a population-based, prospective cohort study of 17708 middle-aged and elderly (main respondents ≥45 years) individuals from 150 counties within 28 provinces in China[17]. The first visit was accomplished in 2011 (visit 1), and subsequently two follow-up visits carried out after that, each nearly two years apart (visit 2 in 2013, visit 3 in 2015). The present analysis was limited to 11847 participants who agreed to perform blood examinations in the visit 1. Out of those participants, 11532 participants who stored whole-blood samples were available for measurement of glycated hemoglobin or fasting glucose. Additionally, 1521 participants were excluded due to incomplete data on Center for Epidemiologic Studies Depression Scale (CES-D) during the 2011-2015 period, as well as 207 main respondents’ spouses who were under 45 years are excluded (Supplementary Fig. 1). Our final sample size was 9804 persons. The study protocol was approved by the institutional review board of Peking University. All participants provided written informed consent.

### Assessment of depressive symptoms

Depressive symptoms were evaluated using the 10-item Center for Epidemiologic Studies Depression Scale (CES-D) [18]. Participants were asked how often they had experienced any of the ten symptoms listed during the past week. The answers for the 10 questions were ranged on a scale from: rarely (0-1 day); to some days (1-2 days); to occasionally (3-4 days); and to most of the time (5-7 days). Scores ranged from 0 for rarely to 3 points for most of the time were assigned for each item and the total scores were calculated and ranged from 0 to 30. CES-D had been shown good validity and reliability in the CHRLS [19]. For the current analysis, missing CES-D exceeding 2 items were excluded. We only included participants who had complete assessments of depression at visits 1, and at least had a one-time assessment at visits 2 or 3.

### Measurement of glycated hemoglobin

A specimen of whole blood was stored at -80 °C in a deep freezer and sent to the Youanmen Center for Clinical Laboratory of Capital Medical University. Total HbA_1_c was measured using standard methods of boronate affinity high performance liquid chromatography. The coefficient of variation of within assay and between assay was 1.90% and 2.10%, respectively. The detection limits of the assay were ranged from 0.0% to 40.0%. Detailed information regarding blood sample collection, processing, transportation, storage, the technicalities of the blood analysis, as well as the quality control and the external quality assessment for the laboratory has been described in the CHALRS Blood Sample Users’ Guide (http://charls.pku.edu.cn/uploads/document/2011-charls-wave1/application/blood_user_guide_en_20140429.pdf).

### Covariates

Sociodemographic information, lifestyle behaviors and medical history were obtained using a face-to-face standard questionnaire and physical examination [17]. The sociodemographic characteristics included age, gender, living area (urban and rural), education and marital status (married and unmarried). Self-reported education was coded as a four levels factor (< primary school, primary school, middle school, and ≥ high school). Each participant was asked two questions: “Have you smoked at least 100 cigarettes in your lifetime?” and “Do you currently smoke cigarettes, even occasionally? The options for possible answer to the questions were either if it was true or not. Smoking status consisted of never, former smoker, or current smoker. Alcohol consumption was determined from one question: “How often did you drink alcoholic beverages in the past?” The answer included never, <1, and ≥1 time/month. Self-reported physician diagnosed diseases included cardiac diseases, stroke, hypertension, diabetes mellitus, dyslipidemia and lung diseases. Antidepressant drugs were recoded and updated during 2011-2015. Trained and certified health professionals conducted a physical examination. Weight was measured using the Omron™ HN-286 scale, and height was measured using Seca™213 stadiometer. Body mass index (BMI) was calculated as weight in kilograms divided by height in meters squared. Obesity was defined as those who had a BMI over 28.0 kg/m^2^ [20]. Blood pressure was measured using the OmronTM HEM-7200 Monitor. Three times blood pressure measurements were taken after the participants were seated, and rested quietly for >5 minutes and the average value was obtained. Cognition scores was consisted of immediate and delayed recall of a 10-word list (20 points), serial 7 subtractions (5 points), orientation (1 point each for year, month, date, day of the week, and season) and drawing a picture (1 point). Then, total scores were calculated, which were ranged from 0 to 31, where higher scores indicated better cognitive function[17]. Overnight fasting blood samples were collected and tested for high-density lipoprotein cholesterol (HDL-C), low-density lipoprotein cholesterol (LDL-C), glucose, high-sensitivity C-reactive protein (hs-CRP), and creatinine by standard methods [21]. The estimated glomerular filtration rate (eGFR) was calculated based on the Chronic Kidney Disease Epidemiology Collaboration equation (CKD-EPI-2009) [22].

### Statistical analysis

Trajectories of depressive symptoms were the main outcomes. Trajectories of depressive symptoms were determined using group-based trajectory modeling (GBTM), an application of finite mixture modeling to map the developmental course of symptoms over time or age[23, 24]. We conducted a censored normal distribution model using Stata traj plugin [25] to estimate the mean trajectories of CES-D scores (measured at year 0, 2, 4) across three visits. The number and the shape of trajectories of depressive symptoms were identified based on a priori. Firstly, the average posterior probabilities of each trajectory group were ≥0.70 and the sample size was ≥5.0% of the population [23]. Secondly, nonsignificant cubic and quadratic terms were removed from trajectories in each model, but linear parameters were retained irrespective of significance. Third, Bayesian Information Criterion (BIC) value and log Bayes factor was calculated and used to determine the best numbers and shapes of the trajectories. The value of log Bayes factor ≥10 was considered a “very strong” indicator where the more complex model was superior to with fewer trajectories[23]. Later, trajectories of depressive symptoms were plotted over follow-up time.

The exposure variable was glycated hemoglobin, and categorized according to quintiles as follows: Quintile 1 (≤4.8%); Quintile 2 (4.9-5.0%); Quintile 3 (5.1-5.2%); Quintile 4 (5.3-5.5%); and Quintile 5 (≥5.6%). Linear trends across the 5 groups were evaluated using the generalized linear model. Multinomial logistic regression was used to calculate the adjusted risk ratios (RR) of decreasing, increasing, and high symptoms of depression compared with low symptoms according to glycated hemoglobin categories, while simultaneously adjusting for confounding covariates. For confounder adjustment, 5 models were evaluated. Model 1 was minimally adjusted for age and gender. Model 2 was adjusted for the additional socio-demographics of marital status, educational level, living area, and health behaviors of smoking and alcohol frequency. Model 3 was further adjusted the baseline health conditions including hypertension, diabetes mellitus, cardiac disease, stroke, dyslipidemia, lung disease, and obesity. Then, we sequentially added other covariates, including cardiac markers (Model 4), baseline cognition scores and antidepressant use over 4 years (Model 5).

In addition, we evaluated the dose-response relationship between glycated hemoglobin, as continuous change, and each trajectory of depressive symptoms using restricted cubic splines[26] with 4 knots corresponding to the 5^th^, 35^th^, 65^th^, and 95^th^ percentiles of glycated hemoglobin distribution. The likelihood-ratio tests were used to assess whether it exited in a nonlinear trend.

Sensitivity analyses were also conducted: 1) changing glycated hemoglobin categories of <4.5%, 4.5-5.0%, 5.1-5.5%, 5.6-6.0%, and >6.0%, respectively; 2) excluding participants who had treatments for depression or diabetes; 3) limited to those participants who were free of depression symptoms (CES-D scores <10[27]) at baseline. We explored the relationship between glycated hemoglobin and incidence of depressive symptoms at visit 3 (year 2015). Lastly, to test whether the associations differed between gender, gender-specific analyses were performed for male and female, separately. Subsequently, a multiplicative term between gender and quantile of HbA_1_c was added in the full-adjusted model and the interaction was tested by a likelihood ratio test.

All analyses were performed with Stata software (version 14.0; Stata Corp., College Station, TX). A two-sided *P* value <0.05 was considered statistically significant.

**Table 1:** Baseline Characteristics of the Study Participants According to the Quintile of Glycated Hemoglobin (HbA_1c_).

Characteristic *	Quintile 1 (≤4.8 %)	Quintile2 (4.9-5.0 %)	Quintile 3 (5.1-5.2 %)	Quintile 4 (5.3-5.5 %)	Quintile 5 (≥5.6 %)	P for Trend
No. of participants	2258	1815	1904	2022	1805	
Glycated hemoglobin, %	4.6 ± 0.2	5.0 ± 0.0	5.1 ± 0.1	5.4 ± 0.1	6.4 ± 1.2	<0.001
Age, yr	58.2 ± 9.4	58.4 ± 9.1	59.0 ± 9.0	59.4 ± 8.8	59.8 ± 8.6	<0.001
Male gender, no. (%)	1088 (48.2)	864 (47.6)	873 (45.9)	925 (45.7)	801 (44.4)	0.008
Married, no. (%)	1997 (88.4)	1607 (88.5)	1700 (89.3)	1787 (88.4)	1609 (89.1)	0.587
Urban, no. (%)	796 (35.3)	623 (34.3)	664 (34.9)	695 (34.4)	744 (41.2)	<0.001
Educational level, no. (%)						<0.001
<Primary school	974 (43.1)	827 (45.6)	895 (47.0)	1012 (50.0)	839 (46.5)	
Primary school	542 (24.0)	379 (20.9)	433 (22.7)	455 (22.5)	406 (22.5)	
Middle school	480 (21.3)	427 (23.5)	391 (20.5)	335 (16.6)	371 (20.6)	
≥High school	262 (11.6)	182 (10.0)	185 (9.7)	220 (10.9)	189 (10.5)	
Smoking status, no. (%)						0.069
Never	1357 (60.1)	1108 (61.0)	1165 (61.2)	1213 (60.0)	1141 (63.2)	
Former smoker	189 (8.4)	157 (8.7)	164 (8.6)	176 (8.7)	171 (9.5)	
Current smoker	712 (31.5)	550 (30.3)	575 (30.2)	633 (31.3)	493 (27.3)	
Alcohol frequency, no. (%)						<0.001
Never	1441 (63.8)	1207 (66.5)	1277 (67.1)	1379 (68.2)	1291 (71.5)	
<1 time/month	199 (8.8)	135 (7.4)	155 (8.1)	151 (7.5)	129 (7.1)	
≥1 time/month	618 (27.4)	473 (26.1)	472 (24.8)	492 (24.3)	385 (21.3)	
Physician diagnosed diseases, no. (%)						
Hypertension	487 (21.6)	433 (23.9)	449 (23.6)	528 (26.1)	578 (32.0)	<0.001
Diabetes mellitus	57 (2.5)	43 (2.4)	43 (2.3)	91 (4.5)	337 (18.7)	<0.001
Cardiac diseases	238 (10.5)	200 (11.0)	241 (12.7)	268 (13.3)	283 (15.7)	<0.001
Stroke	46 (2.0)	32 (1.8)	31 (1.6)	41 (2.0)	52 (2.9)	0.079
Dyslipidemia	178 (7.9)	143 (7.9)	183 (9.6)	188 (9.3)	253 (14.0)	<0.001
Lung diseases	227 (10.1)	174 (9.6)	192 (10.1)	224 (11.1)	179 (9.9)	0.559
BMI, kg/m^2^	23.1 ± 3.8	23.3 ± 3.8	23.4 ± 3.7	23.7 ± 4.2	24.6 ± 4.1	<0.001
Obesity, no. (%)	463 (20.5)	377 (20.8)	379 (19.9)	421 (20.8)	530 (29.4)	<0.001
Systolic BP, mm Hg	129.9 ± 20.4	129.7 ± 20.2	129.8 ± 20.0	130.5 ± 20.8	132.6 ± 19.8	<0.001
Diastolic BP, mm Hg	75.6 ± 11.7	75.7 ± 11.8	75.7 ± 11.5	75.6 ± 11.7	76.8 ± 10.8	0.009
Fasting glucose, mg/dl	100.2 ± 19.4	101.7 ± 17.0	104.3 ± 20.2	107.1 ± 20.0	140.3 ± 65.3	<0.001
LDL Cholesterol, mg/dl	109.9 ± 33.2	115.6 ± 33.0	117.6 ± 35.9	118.2 ± 34.8	122.2 ± 36.6	<0.001
HDL Cholesterol, mg/dl	51.6 ± 15.4	51.9 ± 15.0	50.9 ± 14.5	51.9 ± 16.0	48.5 ± 15.2	<0.001
C-reactive protein, log	0.0 ± 1.1	0.1 ± 1.0	0.1 ± 1.0	0.2 ± 1.1	0.4 ± 1.1	<0.001
Estimated GFR, ml/min/1.73 m^2^	93.5 ± 14.6	93.1 ± 14.1	91.8 ± 14.8	92.2 ± 14.8	90.9 ± 15.2	<0.001
Cognition scores	14.9 ± 5.2	15.0 ± 5.1	14.6 ± 5.2	14.4 ± 5.3	14.4 ± 5.3	<0.001
Antidepressant use ¶, no. (%)	16 (0.7)	8 (0.4)	10 (0.5)	15 (0.7)	14 (0.8)	0.508

* Plus-minus values are means ±SD. Abbreviations: BMI, body mass index; BP, blood pressure; HDL, high-density lipoprotein; LDL, low-density lipoprotein; C-reactive protein was measured in mg/l.

¶ Antidepressant use was updated by every visit from 2011 to 2015.

### RESULTS

Baseline characteristics of the participants according to the quintile of HbA_1c_ were shown in Table 1. The overall mean (±SD) HbA_1c_ of the study population was 5.26 ± 0.81 % (5th to 95th percentile ranged from 4.50 to 6.40 %) (Supplementary Fig. 2). The average concentrations for subjects in the highest quintile (Quintile 5, n=1805), and lowest quintile (Quintile 1, n=2258) were 4.61 ± 0.22%, 6.40 ± 1.25%, respectively. Compared with the lowest quintile of HbA_1c_ (Quintile 1), those with the highest quintile of HbA_1c_ (Quintile 5) were older, and more likely to be female, living in urban areas, more likely to have had a higher education, less likely to be a current smoker or drinker, and more likely to have a higher BMI, blood pressure, fasting glucose, LDL-C, HDL-C and hs-CRP (Table 1). Coexisting conditions were highly prevalent among participants who had the highest levels of HbA_1c_ (Table 1).

During the three visits, data on CES-D scores were available for 9804, 9360, and 9140 participants, respectively. 8696 had data suggesting CES-D scores at all three visits. Firstly, based on a priori, we conducted a GBTM analysis with 3 trajectories (BIC=-85859.06). We found 4 trajectories (BIC=-85666.43) were superior to the model with 3 trajectories. The log Bayes factor was 385.26, revealing that the four-trajectory was the best fit. Although a model with 5 trajectories (BIC=-85577.58) was associated with further improvement in model fit (log Bayes factor=177.7), this model yielded a trajectory with less than 5% of participants (63.25%; 15.69%; 12.23%; 2.28% and 6.55%, respectively). Therefore, four distinct trajectories of depressive symptoms were identified in subsequent analyses. The average posterior probabilities of each trajectory group were 0.90, 0.70, 0.71 and 0.83, respectively.

**Figure 1.**
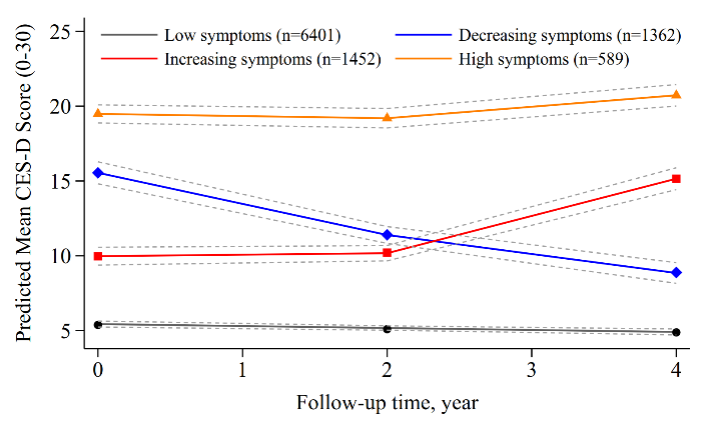
Trajectories of Depressive Symptoms from 2011-2015.

As shown in Figure 1, four depressive symptoms trajectories reflected the patterns of low, decreasing, increasing or high symptoms over 4 years. Trajectory 1, termed “low symptoms” (n=6401, 65.29%), which followed a linear trend (β_0_=5.10, *P*<0.001; β_1_=-0.15, *P*<0.001), represented the individuals who maintained a low CES-D score throughout the follow-up. Trajectory 2, termed “decreasing symptoms” (n=1362, 13.89%) followed a quadratic trend (β_0_=15.50, *P*<0.001; β_1_=-2.43, *P*<0.001; β_2_=0.19, *P*=0.008), in which individuals started at moderately high scores but then remitted. Trajectory 3, termed “increasing symptoms (n=1452, 14.81%)” follows a quadratic trend (β_0_=9.93, *P*<0.001; β_1_=-1.12, *P*<0.001; β_2_=0.61, *P*<0.001) in which CES-D scores increased slowly from year 0 to year 2, and then increased quickly by year 3. Trajectory 4, termed “high symptoms” (n=589, 6.01%) follows a quadratic trend (β_0_=19.49, *P*<0.001; β_1_=-0.67 *P*<0.001; β_2_=0.25 *P*<0.001), in which individuals maintained high scores throughout.

**Table 2:** Risk Ratios for the Association Between Quintile of Glycated Hemoglobin (HbA_1_c) and Risk of Trajectories of Depressive Symptoms *

	Low symptoms (n=6401)	Decreasing symptoms (n=1362)	Increasing symptoms (n=1452)	High symptoms (n=589)
Model 1: Adjusted for age and gender				
Quintile 1		1.00 (Reference)	1.00 (Reference)	1.00 (Reference)
Quintile 2		0.90 (0.75-1.09)	1.07 (0.89-1.28)	0.85 (0.64-1.13)
Quintile 3		1.05 (0.88-1.25)	1.02 (0.85-1.22)	1.08 (0.83-1.41)
Quintile 4		0.97 (0.81-1.16)	1.28 (1.08-1.52)	1.00 (0.77-1.30)
Quintile 5		1.08 (0.90-1.30)	1.18 (0.99-1.42)	1.29 (1.00-1.67)
Model 2: Adjusted for demographics a and health behaviors b				
Quintile 1		1.00 (Reference)	1.00 (Reference)	1.00 (Reference)
Quintile 2		0.89 (0.74-1.07)	1.05 (0.88-1.27)	0.83 (0.62-1.1)
Quintile 3		1.04 (0.87-1.25)	1.01 (0.84-1.22)	1.08 (0.83-1.41)
Quintile 4		0.94 (0.78-1.13)	1.26 (1.06-1.50)	0.97 (0.74-1.26)
Quintile 5		1.12 (0.93-1.35)	1.22 (1.02-1.47)	1.37 (1.05-1.77)
Model 3: Adjusted for demographics a, health behaviors b and baseline health conditions ^c^				
Quintile 1		1.00 (Reference)	1.00 (Reference)	1.00 (Reference)
Quintile 2		0.89 (0.73-1.07)	1.05 (0.87-1.26)	0.83 (0.62-1.11)
Quintile 3		1.03 (0.86-1.24)	1.00 (0.84-1.21)	1.08 (0.82-1.41)
Quintile 4		0.92 (0.76-1.10)	1.24 (1.04-1.47)	0.93 (0.71-1.22)
Quintile 5		1.07 (0.88-1.29)	1.18 (0.98-1.42)	1.28 (0.97-1.68)
Model 4: Adjusted for demographics a, health behaviors b, baseline health conditions c and cardiac marker d				
Quintile 1		1.00 (Reference)	1.00 (Reference)	1.00 (Reference)
Quintile 2		0.88 (0.73-1.06)	1.05 (0.87-1.26)	0.84 (0.63-1.13)
Quintile 3		1.03 (0.86-1.24)	1.00 (0.83-1.20)	1.12 (0.85-1.46)
Quintile 4		0.90 (0.75-1.08)	1.22 (1.02-1.46)	0.97 (0.74-1.27)
Quintile 5		1.05 (0.85-1.28)	1.14 (0.94-1.39)	1.46 (1.09-1.95)
Model 5: Adjusted for demographics a, health behaviors b, baseline health conditions c, cardiac marker d, antidepressant use and cognition scores				
Quintile 1		1.00 (Reference)	1.00 (Reference)	1.00 (Reference)
Quintile 2		0.89 (0.73-1.08)	1.05 (0.87-1.27)	0.84 (0.63-1.13)
Quintile 3		1.02 (0.85-1.23)	0.99 (0.82-1.20)	1.10 (0.84-1.45)
Quintile 4		0.89 (0.74-1.07)	1.21 (1.01-1.44)	0.95 (0.72-1.24)
Quintile 5		1.01 (0.82-1.25)	1.12 (0.92-1.36)	1.39 (1.04-1.86)

* Data was reported as risk ratios (95%CI) from multinomial logistic regression.

a Demographic factor were age, gender, marital status, educational level, and living area.

b Health behaviors consisted of smoking, and alcohol frequency.

c Baseline health conditions included hypertension, diabetes mellitus, cardiac disease, stroke, dyslipidemia, lung disease, and obesity.

d Cardiac marker consisted of systolic blood pressure, fasting glucose, LDL cholesterol, HDL cholesterol, log-transformed C-reactive protein and eGFR.

**Table 3:** Risk of Incident Increasing and High Depressive Symptoms Associated with Glycated Hemoglobin Level.

Glycated hemoglobin, %	Risk Ratios (95% CI) *	
	Increasing symptoms	High symptoms
4.5	1.01 (0.89-1.14)	1.10 (0.91-1.33)
4.8	0.99 (0.96-1.03)	1.00 (0.95-1.06)
5.0	1.00	1.00
5.2	1.04 (0.99-1.08)	1.08 (1.01-1.15)
5.5	1.10 (0.99-1.21)	1.22 (1.03-1.43)
5.8	1.14 (1.00-1.30)	1.30 (1.05-1.61)
6.0	1.16 (1.01-1.33)	1.33 (1.05-1.68)
6.2	1.18 (1.02-1.36)	1.35 (1.06-1.72)
6.5	1.20 (1.04-1.40)	1.36 (1.04-1.77)

* Adjusted for age, gender, marital status, educational level, living area, smoking, alcohol frequency, hypertension, diabetes mellitus, cardiac disease, stroke, dyslipidemia, lung disease, obesity, systolic blood pressure, fasting glucose, LDL cholesterol, HDL cholesterol, log-transformed C-reactive protein, estimated GFR, antidepressant use and cognition scores.

During a 4 year of follow-up, 130 (5.76%), 91(5.01%), 120 (6.30%), 116 (5.74%) and 132 (7.31%) subjects developed high depressive symptoms across the quintile of HbA_1c_, respectively. Corresponding increasing depressive symptoms were 305 (13.51%), 266 (14.66%), 261 (13.71%), 342 (16.91%) and 278 (15.40%). Individuals with a high HbA_1c_ were significantly more likely to have increasing and high depressive symptoms compared with their counterparts with low HbA_1c_ (Table 2). The RR of highest HbA_1c_ of high depressive symptoms was 1.37 (95% CI: 1.05-1.77) as compared with those with the lowest levels of HbA_1c_ after adjusted for demographics and health behaviors variables. In addition, we further adjusted for systolic blood pressure, fasting glucose, LDL cholesterol, HDL cholesterol, log-transformed hs-CRP, eGFR, antidepressant use and cognition scores, the multivariate-adjusted risk ratios of high depressive symptoms remained significant (RR:1.39, 95% CI:1.04-1.86). Meanwhile, the risk for increasing symptoms of depression of highest HbA_1c_ (Quintile 5) still was not statistically significant compared with Quintile 1 (adjusted RR: 1.12, 95% CI: 0.92-1.36). The corresponding Quintile 4 group was at high risk of increasing symptoms of depression (adjusted RR: 1.21, 95% CI: 1.01-1.44).

**Figure 2.**
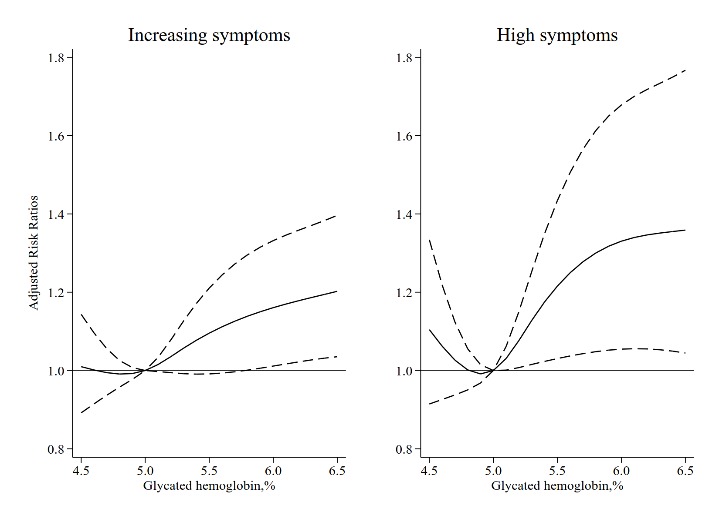
Risk of Incident Increasing and High Depressive Symptoms Associated with Glycated Hemoglobin Level. Solid curve represents estimates of the risk ratios. The dashed lines represent pointwise 95% confidence intervals. HbA_1_c of 5% was used as the reference because it approximated the median values. The graphs are truncated at the 5^th^ and 95^th^ percentiles.

We also used restricted cubic splines to estimate the trend in the risk for increasing and high depressive symptoms. The spline function for HbA_1c_ confirmed the nonlinear, J-shaped relationship with the risk of high depressive symptoms. Whereas the association of HbA_1c_ concentration with increasing depressive symptoms appeared to be linear throughout the range from the 5^th^ to the 95^th^ percentile of HbA_1c_ (Fig. 2). For instance, for a HbA_1c_ levels of 6.5% compared to 5%, the adjusted risk ratios for increasing and high depressive symptoms was 1.20 (95% CI: 1.04-1.40) and 1.36 (95% CI: 1.04-1.77), respectively (Table 3).

Our results were robust in various sensitivity analyses. Firstly, results from this analysis using an alternative HbA_1_c category was consistent with the previous results; in particular, high levels of HbA_1_c (>6.0%) was still associated with a significantly increased risk of increasing (RR: 1.36, 95% CI: 1.03-1.79) and high (RR: 1.73, 95% CI: 1.14-2.63) symptoms of depression. (Supplementary Fig. 3). Secondly, the results remained essentially unchanged after excluding these individuals who had treatments for diabetes or depression (Supplementary Table 1). Thirdly, we excluded those who had depressive symptoms at baseline (n=3715), as well as participants with missing data of CES-D in visits 3 (year 2015) (n=422). The final analytical sample was limited to 5667 persons. During a 4-year follow-up, 1281 (n=22.60%) incident depressive symptoms were identified. The adjusted risk ratios of the Quintile 4 group (ranged from 5.3% to 5.5%) compared with the lowest quantile group of HbA_1_c was 1.29 (95% CI: 1.12-1.49). Corresponding risk ratios for the highest HbA_1_c quantile was 1.12 (95% CI: 0.95-1.33) (Supplementary Table 2). Lastly, for male, the risk ratios of high symptoms for participants in the second and top HbA_1_c quintile were 0.91 (95%CI: 0.55-1.51) and 1.20 (95% CI: 0.71-2.03), respectively. Corresponding risk ratios for female were 0.81 (95% CI: 0.56-1.17) and 1.49 (95% CI: 1.04-2.11). We found a marginal difference in the association of quantile of HbA1c with different trajectories of depressive symptoms between male and female (Likelihood-ratio test: χ^2^=18.48, *P* for interaction=0.102) (Supplementary Table 3).

### DISCUSSION

We observed four trajectories of depressive symptoms characterized by low, decreasing, increasing, and high symptoms in a large prospective cohort of 9804 participants during a 4-year follow-up. Individuals with the highest quintile of HbA_1c_ (≥5.6%) had the higher risk of developing increasing and high depressive symptoms compared with those with the lowest quintile (≤4.8%). Moreover, we observed a J-shaped relation between HbA_1c_ and the risk of high depressive symptoms, with the lowest risk at a HbA_1c_ of 5.0% from restricted cubic splines analysis.

To the best of our knowledge, four trajectories of depressive symptoms were firstly identified in the middle-aged and older Chinese adults. Previously, three trajectories of depressive symptoms (consistently minimal, moderate and increasing, and high and increasing symptoms) were identified with four CES-D measures from baseline to year 5 among 2488 older adults from the Health ABC prospective cohort study [28]. Five trajectories of depressive symptoms (low, decreasing, remitting, increasing, and high depressive symptoms) were also observed in the Rotterdam Study [5, 29]. Differences of shape and the number of trajectories of depressive symptoms were was due to the time of CES-D measurements as well as the follow-up time. We noted that, in the current study that, among the 9804 participants aged ≥60 years, 6.01% of individuals had the increasing and high-stable symptoms of depression respectively. Prospective cohort studies had demonstrated that individuals with increasing or high-stable symptoms of depression had a higher risk of dementia [5, 28]. Therefore, physicians should pay more attention to these individuals and treat positively depressive symptoms in order to reduce the burden of dementia in the clinical practice.

There has been a sizable literature that reported on the association between diabetes and elevated depressive symptoms [8, 13, 15, 16, 30]; however, research related to the association between HbA_1c_ and the incidence of depressive symptoms is limited. For example, no significant association was observed in the English Longitudinal Study of Aging after adjusted full covariates (OR: 1.08, 95%: 0.91-1.29 per 1% HbA_1c_ increment) (Hamer et al., 2011). In addition, in the Health, Aging, and Body Composition Study, HbA_1c_ ≥7% did not increased the risk of depressive symptoms (RR: 1.21, 95% CI: 0.94-1.55), whereas high HbA_1c_ was associated with a two-fold risk of recurrent depressive symptoms (RR: 2.10, 95% CI: 1.36-3.22) compared with HbA_1c_ <7% during a mean follow-up of 5.9 years among older person aged from 70-79 years. This indicated that single measures of depressive symptoms may be inaccurate, and suggests that depressive symptoms seem to fluctuate over time. Additionally, a recent study conducted by *Ravona-Springer et al.* revealed that the long-term variability in HbA_1c_ was associated with more subsequent depressive symptoms [31]. This was consisted with our findings. However, in this study[31], depressive symptoms was only measured using Geriatric Depression Scale (GDS-15) at baseline and the impact of the variability in HbA_1c_ on the incidence of depressive symptoms was unclear. In our study, HbA_1c_ was only available at baseline, and the association between the long-term change of HbA_1c_ and the longitudinal pattern of depressive symptoms is required to be validated in a large population-based longitudinal study.

Another interesting finding was that HbA_1c_ also was associated with a high risk of an increasing depressive symptoms trajectory. Previous studies have shown that poor glycemic control at baseline was associated with increased risk for the incidence of depressive symptoms [15, 16, 32]. To confirm the findings, we also repeated the analysis among the non-depressed individuals and examined the role of HbA_1c_ as a risk for new-onset depressive symptoms over a 4-year follow-up period. However, the highest risk of depression was observed at HbA_1_c levels of 5.3-5.5% (Quintile 4), rather than the highest Quintile (≥5.6 %). This finding had some discrepancies with previously findings related to the threshold of HbA_1_c [16, 33], which may due to the difference in the study design, the definition of increasing symptoms of depression, the distribution and categorization of HbA_1_c in the study population. Overall, we found HbA_1_c was linked to increasing symptoms of depression. Nonetheless, more research is needed to examine this association. Increasing symptoms of depression was meant to be emphasized, which also reflected glycemic control [32, 34] and increased the risk of diabetes [35], dementia [5, 28] and all-cause mortality [29].

Our findings further demonstrated that baseline HbA_1c_ was associated with increasing and high depressive symptoms. Results were not affected by further adjustment for sociodemographic, history of diabetes, fasting glucose, and other confounders that related to depressive symptoms. In addition, depressive symptoms and cognitive decline were coexisting in late-life [36]. The relationship was robust after adjusting for cognitive scores. To account for bias due to use of antidepressant and/or antidiabetic medication, we repeated the analysis after excluding those relevant participants. The results remained considerably unaltered. Lastly, a slight stronger association between HbA_1_c and depressive symptoms was observed among female, however, gender differences were not statistically significant (*P* for interaction = 0.102). This may be due to different levels of estrogen [37]. The findings were consistent with a previously study conducted in the Nurses’ Health Study cohort [38].

The mechanisms underlying the relationship of HbA_1c_ with subsequent risks of high symptoms of depression remain unclear. Firstly, the “vascular depression” hypothesis may explain the relationship [39]; HbA_1c_ reflects the glycemic control for diabetes patients, and brain vasculature and the functional area may be mostly vulnerable to worse glycemic control [40].This may be related to the process of depression. Secondly, late-life risk factors, including smoking, obesity, and chronic diseases were both associated with high HbA_1c_ and depressive symptoms [41].

Several limitations in our study should be noted. First, although HbA_1c_ predicted the incidence of high depressive symptoms, the causal relationship between HbA_1c_ and depressive symptoms has not been fully established. Due to this study being observational in nature, residual confounding could not be fully eliminated. Besides, the association was based on single glycated hemoglobin measurements at the baseline as well as the fact that the relationship of variability in HbA_1c_ over years, with subsequent increasing and high-stable depressive symptoms could not be examined. Major strengths of our study were that it was the largest, nationwide population-based cohort study, as well it obtained a high response rate in CES-D measures (89% for all three visits). Moreover, the study design of CHARLS was referred to the Health and Retirement Study [42] and comprehensive and rigorous measurements of risk factors were collected. This gave us an opportunity to adjust more potential confounding variables.

### Conclusions

In summary, different trajectories of depressive symptoms were identified by repeated measures of CES-D from China Health and Retirement Longitudinal Study. Our study findings suggest that clinicians should be aware of the increased risk of increasing and elevated high depressive symptoms for individuals with high HbA_1c_, irrespective of the history of diabetes.

## Supplementary data

The Supplemenantry data can be found online at:www.aginganddisease.org/EN/10.14336/AD.2018.0410
