# Supplementary material for: Prospective Study of Glycated Hemoglobin and Trajectories of Depressive Symptoms: The China Health and Retirement Longitudinal Study
Source: Aging Dis. 2019 Apr 1;10(2):249–57. doi: 10.14336/AD.2018.0410 (PMC6457062; doi:10.14336/AD.2018.0410)
Supplement: Supplementary file 1 — The Supplemenantry data can be found online at:www.aginganddisease.org/EN/10.14336/AD.2018.0410 [file ad-10-2-249-s.pdf]

SUPPLEMENTARY DATA

Prospective Study of Glycated Hemoglobin and Trajectories of Depressive Symptoms: The China Health and Retirement Longitudinal Study

Haibin Li[1,2], Anxin Wang[1,2], Wei Feng[1,2], Deqiang Zheng[1,2], Qi Gao[1,2], Lixin Tao[1,2], Jin Guo[3], Xiaonan Wang[1,2], Xia Li[4], Wei Wang[5], Xiuhua Guo[1, 2*]

[1]Department of Epidemiology and Health Statistics, School of Public Health, Capital Medical University, Beijing, China. [2]Beijing Municipal Key Laboratory of Clinical Epidemiology, Capital Medical University, Beijing, China. [3]Greenwood Medical Company, 300 Highway Burwood, Melbourne, Melbourne, Victoria, Australia. [4]Department of Mathematics and Statistics, La Trobe University, Victoria, Australia. [5]Global Health and Genomics, School of Medical Sciences and Health, Edith Cowan University, Perth, Western Australia, Australia.

© 2018. Li H et al. Published online at http://www.aginganddisease.org/EN/10.14336/AD.2018.0404

SUPPLEMENTARY DATA

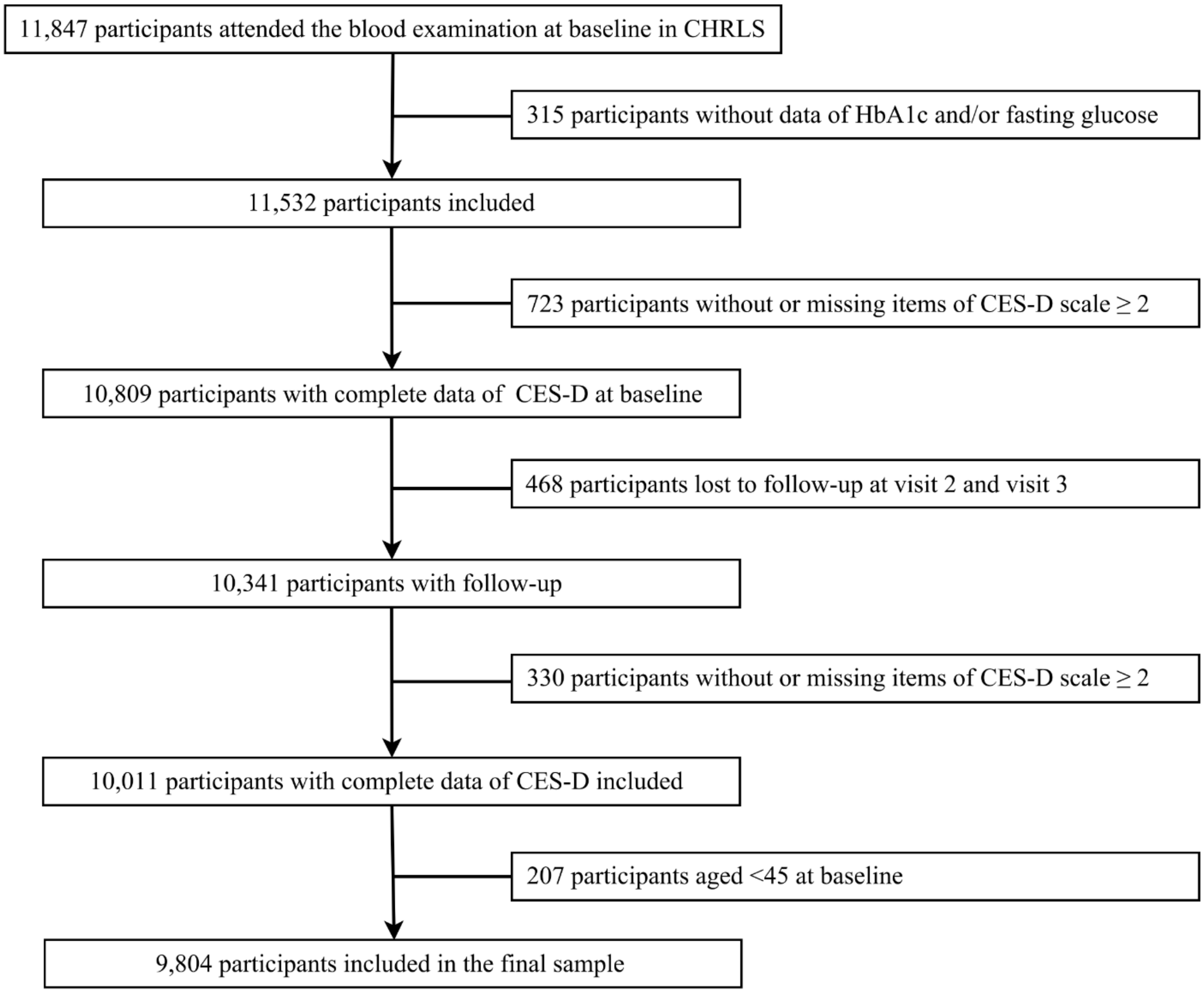

Supplementary Figure 1. Flowchart of Study Profile.

© 2018. Li H et al. Published online at http://www.aginganddisease.org/EN/10.14336/AD.2018.0404

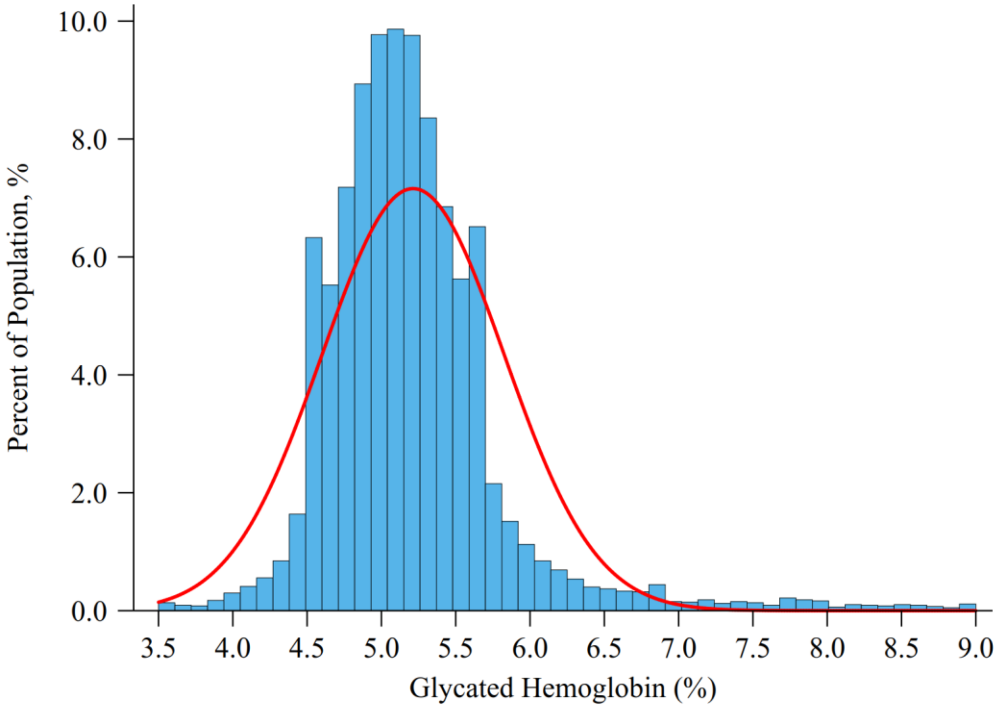

Supplementary Figure 2. Histogram of Serum glycated hemoglobin Concentrations. Red Solid curve denotes the normality plot.

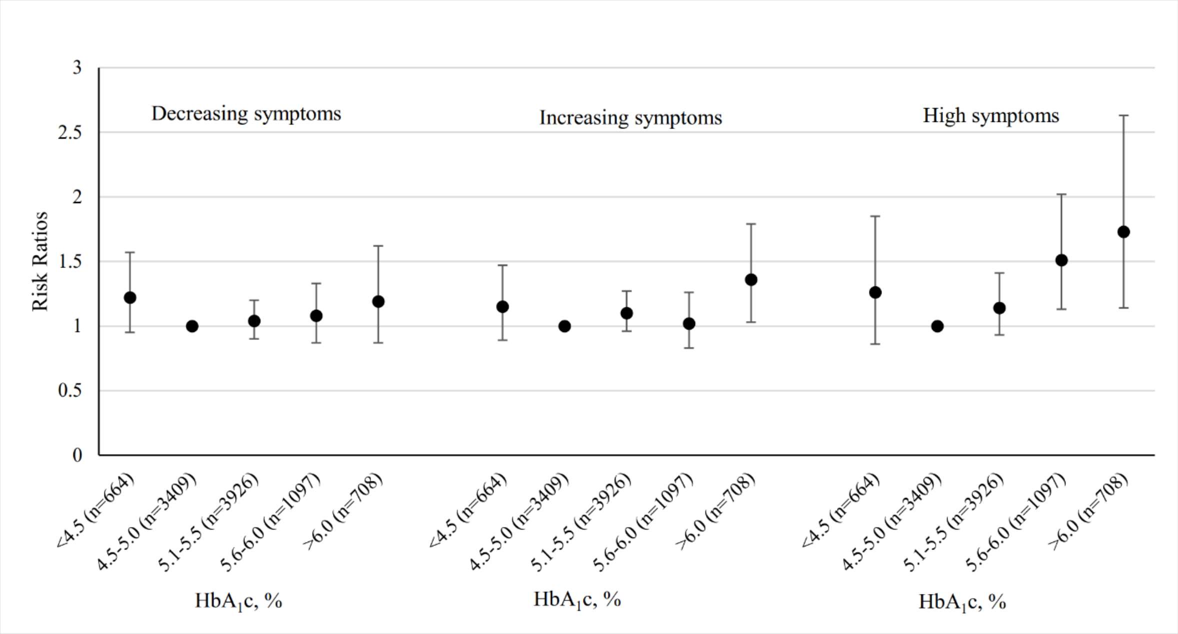

Supplementary Figure 3. The risk ratios of decreasing, increasing and high depressive symptoms by using an alternative HbA$_1$c category (<4.5%, 4.5-5.0%, 5.1-5.5%, 5.6-6.0%, and >6.0%).

© 2018. Li H et al. Published online at http://www.aginganddisease.org/EN/10.14336/AD.2018.0404

Supplementary Table 1. Sensitivity Analyses for the Association Between Quintile of Glycated Hemoglobin (HbA_{1C}) and Risk of Trajectories of Depressive Symptoms*

	Low symptoms	Decreasing symptoms	Increasing symptoms	High symptoms
Excluding participants who had antidepressant use (n=63) ¶				
Quintile 1		1.00 (Reference)	1.00 (Reference)	1.00 (Reference)
Quintile 2		0.88 (0.73-1.07)	1.05 (0.87-1.27)	0.86 (0.64-1.15)
Quintile 3		1.01 (0.84-1.22)	1.00 (0.83-1.20)	1.12 (0.85-1.47)
Quintile 4		0.89 (0.73-1.07)	1.20 (1.01-1.44)	0.93 (0.71-1.22)
Quintile 5		1.02 (0.83-1.26)	1.12 (0.92-1.37)	1.38 (1.03-1.85)
Excluding participants who had treatments for diabetes (n=394) †				
Quintile 1		1.00 (Reference)	1.00 (Reference)	1.00 (Reference)
Quintile 2		0.88 (0.73-1.08)	1.07 (0.89-1.29)	0.86 (0.64-1.16)
Quintile 3		1.03 (0.86-1.24)	0.99 (0.82-1.20)	1.15 (0.87-1.51)
Quintile 4		0.89 (0.74-1.08)	1.23 (1.03-1.47)	0.96 (0.73-1.27)
Quintile 5		1.08 (0.88-1.34)	1.11 (0.91-1.37)	1.44 (1.07-1.94)
Excluding participants who had treatments for depression and/or diabetes (n=453) ‡				
Quintile 1		1.00 (Reference)	1.00 (Reference)	1.00 (Reference)
Quintile 2		0.88 (0.72-1.07)	1.07 (0.89-1.29)	0.88 (0.65-1.18)
Quintile 3		1.02 (0.85-1.23)	1.00 (0.83-1.21)	1.16 (0.88-1.53)
Quintile 4		0.89 (0.74-1.08)	1.23 (1.02-1.47)	0.94 (0.71-1.25)
Quintile 5		1.09 (0.88-1.35)	1.12 (0.92-1.38)	1.43 (1.06-1.93)

*Data was reported as risk ratios (95%CI) from multinomial logistic regression.
¶ Model was adjusted for demographics [a], health behaviors [b], baseline health conditions [c], cardiac marker [d], and cognition scores.
† Model was adjusted for demographics [a], health behaviors [b], baseline health conditions (excluding treated diabetes) [c], cardiac marker [d], antidepressant use and cognition scores.
‡ Model was adjusted for demographics [a], health behaviors [b], baseline health conditions [c], cardiac (excluding treated diabetes) marker [d], and cognition scores.

Supplementary Table 2. Risk Ratios for the Association Between Quintile of Glycated Hemoglobin (HbA_{1C}) and Risk of Incident Depressive Symptoms During 4-year of follow-up Among Nondepressed Individuals at Baseline*

	No. cases	Crude IRRs	Adjusted IRRs ¶
Quintile 1 (n=1325)	259	1.00 (Reference)	1.00 (Reference)
Quintile 2 (n=1080)	246	1.17 (1.00-1.36)	1.16 (1.00-1.35)
Quintile 3 (n=1116)	247	1.13 (0.97-1.32)	1.11 (0.96-1.30)
Quintile 4 (n=1174)	309	1.35 (1.16-1.56)	1.29 (1.12-1.49)
Quintile 5 (n=972)	220	1.16 (0.99-1.36)	1.12 (0.95-1.33)

*Data was reported as incidence rate ratio (95%CI) from modified Poisson regression models with a robust error variance.
¶Model was adjusted for demographics [a], health behaviors [b], baseline health conditions [c], cardiac marker [d], antidepressant use and cognition scores (Model 5) plus baseline CES-D scores.
